# Energetic diversity in retinal ganglion cells is modulated by neuronal activity and correlates with resilience to degeneration

**DOI:** 10.21203/rs.3.rs-5989609/v1

**Published:** 2025-03-12

**Authors:** Zelun Wang, Christopher Zhao, Shelly Xu, Sean McCracken, Rajendra S. Apte, Philip R. Williams

**Affiliations:** 1 John F. Hardesty, MD Department of Ophthalmology and Visual Sciences, Washington University School of Medicine, St. Louis, MO 63110, USA; 2 Graduate Program in Neuroscience, Washington University School of Medicine, St. Louis, MO 63110, USA; 3 Medical Scientist Training Program, Washington University School of Medicine, St. Louis, MO 63110, USA; 4 Department of Developmental Biology, Washington University in St. Louis School of Medicine, St. Louis, Missouri, USA; 5 Department of Medicine, Washington University in St. Louis School of Medicine, St. Louis, Missouri, USA; 6 Department of Neuroscience, Washington University School of Medicine, St. Louis, MO 63110, USA; 7 Hope Center for Neurological Disorders, Washington University School of Medicine, St. Louis, MO 63110, US

## Abstract

Neuronal function requires high energy expenditure that is likely customized to meet specific signaling demands. However, little is known about diversity of metabolic homeostasis among divergently-functioning types of neurons. To this end, we examined retinal ganglion cells (RGCs), a population of closely related, yet electrophysiologically distinct excitatory projection neurons. Using *in vivo* 2-photon imaging to measure ATP with single cell resolution, we identified differential homeostatic energy maintenance in the RGC population that correspond to distinct RGC types. In the presence of circuit activity, the most active RGC type (Alpha RGCs), had lower homeostatic ATP levels than other types and exhibited the greatest magnitude of ATP decline when ATP synthesis was inhibited. By simultaneously manipulating circuit activity and mitochondrial function, we found that while oxidative phosphorylation was required to meet ATP demands during circuit activity, it was expendable to maintain resting ATP levels. We also examined ATP signatures associated with survival and injury response after axotomy and report a correlation between low homeostatic ATP and increased survival. In addition, we observed transient ATP increases in RGCs following axon injury. Together, these findings identify diversity of energy handling capabilities of dynamically active neurons with implications for neuronal resilience.

## INTRODUCTION

Our nervous system is energetically demanding as it consumes a disproportionate amount of fuel relative to the rest of our bodies, and neurons consume most of that energy quota ^1–3^. Despite or perhaps because of the extreme energy expenditure of neurons, evolutionary pressures have forced energetic savings where possible into the properties of individual neurons, circuit wiring patterns, and glial/vascular support processes ^4,5^. Therefore, metabolic specializations likely exist across different types of neurons to best accommodate their unique signaling requirements. While progress has been made in understanding the metabolic characteristics of neurons in general and their interplay with glial cells, energetic diversity across neuronal populations is much less understood. Thus, we were motivated to query the intrinsic differences in energetic properties across physiologically diverse but otherwise closely related neurons as it relates to their signaling function.

In this study we examine the energetics of retinal ganglion cells (RGCs), a population of excitatory projection neurons that share the same milieu, and are closely related in developmental origins as well as general synaptic and morphological features. Yet RGCs are comprised of electrophysiologically divergent types, thus allowing direct comparison of their energetic properties. We focus on three RGC type families, Alpha RGCs (αRGCs), intrinsically photosensitive RGCs (ipRGCs) and ON-OFF direction selective RGCs (ooDSGCs) because they represent a range of signaling properties that may exhibit differential homeostatic and activity-related energetic characteristics ^6–9^. Importantly, these families also contrast in their resilience to neurodegeneration, allowing for inferences of the relationship between energetic homeostasis and RGC resiliency in the face of injury ^10–15^.

Previous studies of RGC metabolism have relied on whole retinal tissue or the enriched total RGC population ^16–20^. Although such results have been insightful, they are limited by the small proportional representation of RGCs in the whole retina (1% of cells) and the inability to examine differences among RGCs ^21^. Furthermore, the complement of vasculature, glia, and interstitial fluid composition in the retina likely creates a metabolic microenvironment difficult to faithfully recapitulate *ex vivo* ^22^. To examine metabolic heterogeneity among RGCs in their native environment, we used transpupillary 2-photon *in vivo* imaging of ATeam, a well-established genetically-encoded fluorescent biosensor that measures ATP concentration ^23^, to measure ATP of RGCs at individual-cell resolution ^24,25^. Correlating this *in vivo* metabolic imaging with immunostaining, we characterize ATP heterogeneity in the RGC population and identify type-specific ATP differences. Through intraocular injection of metabolic inhibitors under varying activity states, we also describe the role of activity in energy consumption *in vivo* and identify metabolic requirements for maintaining energetic homeostasis during activity-related ATP consumption. Finally, we induced neurodegeneration using an optic nerve crush model and examined energetic features that correlate with survival, as well as observed the longitudinal energetic response to degenerative conditions. Through studying these RGCs, we describe heterogeneity in ATP metabolism of a population of neuron types in their shared native microenvironment, characterize the role of activity and OXPHOS in ATP homeostasis, and identify energetic responses to neurodegeneration along with features of resilient neurons.

## RESULTS

### ATP levels vary across RGC types

**Figure 1.**
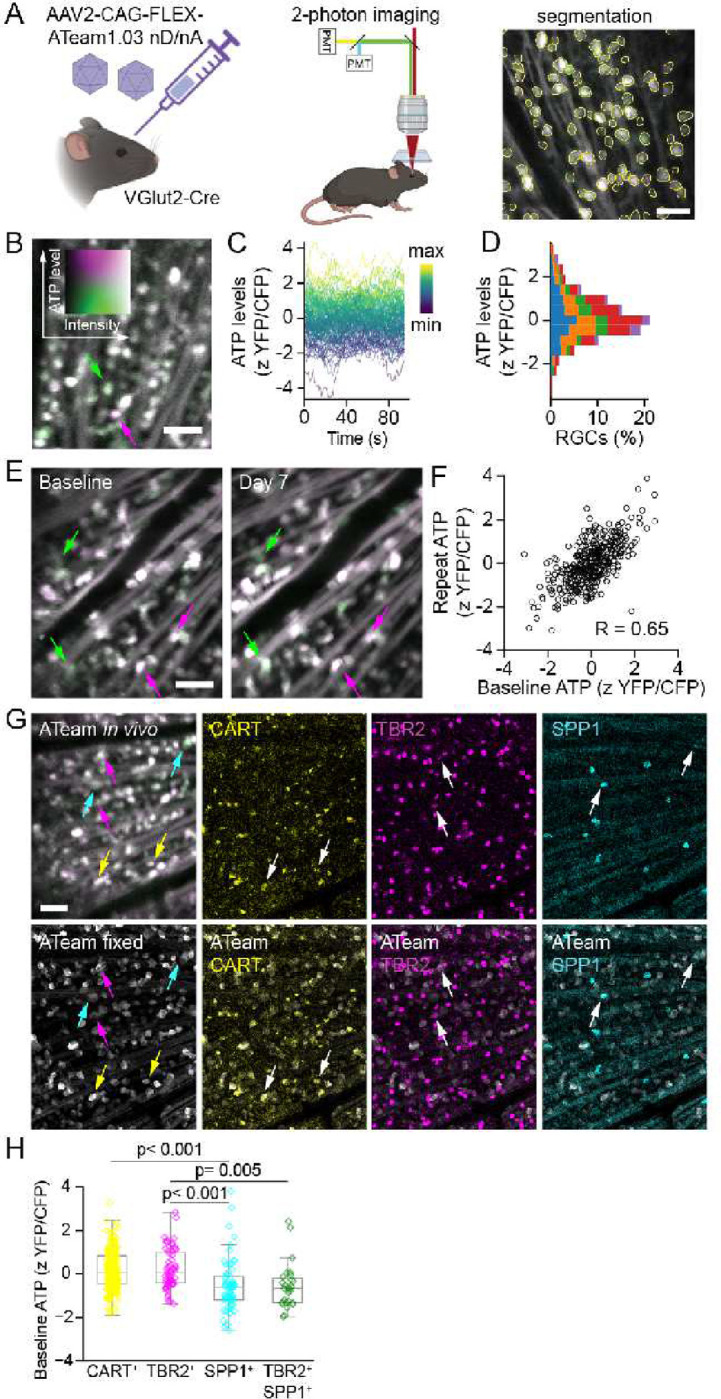
ATP heterogeneity across RGCs. (A) ATeam was expressed in RGCs by intraocular injection of a Cre-dependent AAV expression vector into VGlut2-Cre mice. Transpupillary *in vivo* 2-photon imaging measured Cp173-mVenus (YFP) and mseCFP (CFP) intensity of ATeam in RGC somas segmented with Cellpose. (B) *In vivo* 2-photon average intensity projection (aip) of ATeam time lapse image series. YFP and CFP pseuodocolored magenta and green respectively throughout. Arrows indicate RGCs with lower (green) and higher (magenta) ATP levels. (C) Traces of single RGC ATP levels tracked in an example retina, 20 frame moving average graphed throughout. zScores are pseudocolored from minimal (blue) to maximal (yellow) by average over the sample trace. (D) Histogram of ATP level distributions across multiple retinas (n=537 RGCs from 5 retinas). Each retina is represented by a unique color. (E) *In vivo* 2-photon aips of the same retina imaged 7 days apart. Arrows indicate the same example RGCs. (F) Scatterplot of ATP levels measured in RGCs initially and 7 days later. Each point represents a single RGC. R = Pearson’s correlation coefficient throughout (n=381 RGCs from 7 retinas). (G) *In vivo* 2-photon aip of ATeam (upper left). Confocal maximum intensity projection (mip) of fixed YFP signal from ATeam (lower left), and indicated RGC type marker immunostaining for CART (yellow), TBR2 (magenta) and SPP1 (cyan). Arrows indicate example RGCs and match type coloring on left panels. (H) Scatter and boxplots of ATP levels by RGC types. ANOVA with posthoc Tukey’s test (n=349 RGCs from 5 retinas). Scale bars = 50 μm. Diagrams created with BioRender.

To directly measure ATP levels in individual RGCs, we expressed ATeam1.03-nD/nA (referred to as ATeam throughout), a genetically-encoded FRET-based biosensor, in murine RGCs by delivering a Cre-dependent AAV2 expression construct into the eyes of VGlut2-Cre transgenic mice ^26,27^. This strategy labels RGCs and horizontal cells in the retina; since horizontal cells in the retinal inner nuclear layer are deep to the RGCs, they were not included for imaging ^25^. We measured ATP levels (YFP/CFP ∝ [ATP]) by time-lapse *in vivo* imaging through the pupil using 2-photon microscopy (Fig1A). High-throughput quantification was accomplished by neural network-based segmentation of RGC somas using Cellpose, run on a custom model trained on our dataset ^28^. ATP levels varied across the RGC population, with somas exhibiting a range of YFP/CFP values distributed normally within each retina (Fig1B–D). To verify that ATP differences within a population represent true heterogeneity characteristic to individual RGCs, we performed repeat imaging of the same cells one week later, which showed consistent baseline ATP differences (*R* = 0.65) (Fig1E–F).

To determine whether baseline ATP heterogeneity was related to RGC type, we measured ATP *in vivo* and then collected retinas to perform immunostaining of the imaged regions for RGC type markers. We labeled αRGCs, ipRGCs, and ooDSGCs using antibodies for SPP1, TBR2 and CART, respectively (Fig1G) ^12,29,30^. Landmark-based deformable alignment of the *in vivo* and immunostained images using BigWarp enabled efficient matching of ATP measurements to RGC type ^31^. We found that αRGCs had lower baseline ATP compared to ipRGCs and ooDSGCs (Fig1H). αRGCs are comprised of four subtypes corresponding to their light response properties (ON- or OFF- depolarizing and transient vs sustained firing). Importantly, sustained αRGCs demonstrate more activity than their transient counterparts; thus we examined if heterogeneity in ATP levels may be present within the αRGC family ^6^. One αRGC subtype, the αON-sustained RGC (αON-S), is also the M4 subtype of ipRGC, and expresses both SPP1 and TBR2 ^32^. We found that baseline ATP levels in αON-S RGCs were not statistically different compared to other αRGCs, but were significantly lower than for the other ipRGC subtypes (Fig1H). Taken together, our results demonstrate that homeostatic ATP levels can differ between RGCs within the same microenvironment *in vivo*, and that these differences are in part related to RGC type.

### αRGCs exhibit the greatest ATP depletion under mitochondrial inhibition

**Figure 2.**
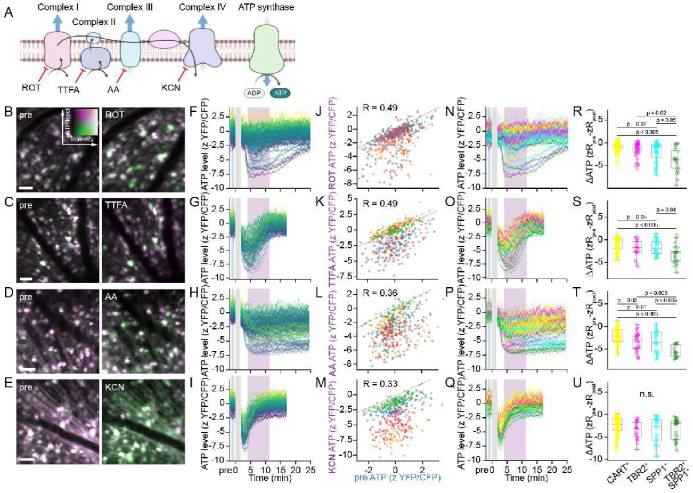
RGCs are differentially affected by mitochondrial inhibition. (A) Schematic of ETC inhibitors and their targets. (B-E). *In vivo* 2-photon aips of ATeam in the same retina imaged before and after intravitreal injection of the indicated pharmacological inhibitor. Scale bars = 50 μm. (F-I). Traces of single RGC ATP levels tracked in an example retina in response to the indicated ETC inhibitor. Each line indicates an individual RGC. zScores are pseudocolored from minimal (blue) to maximal (yellow) according to the values in the pre trace. (J-M) Scatterplot of ATP levels in RGCs comparing pre and post injection of the indicated ETC inhibitor. Each point represents an RGC, and each color indicates a retina (ROT n=264 RGCs from 5 retinas, TTFA n = 212 RGCs from 4 retinas, AA n=220 RGCs from 4 retinas, KCN n=218 RGCs from 4 retinas). (N-Q) Example traces of ATP levels in (F-I) pseudocolored by RGC type according to *post hoc* immunostaining as performed in Figure 1F. Yellow = CART^+^, magenta = TBR2^+^, cyan = SPP1^+^, green = TBR2^+^SPP1^+^ and gray indicates negative for all markers. (R-U) Scatter and boxplots of post injection changes in ATP levels from pre (blue box in N-Q) for indicated ETC inhibitor (purple box in N-Q). ANOVA with posthoc Tukey’s test.

To further characterize differential energy maintenance across RGCs, we investigated the effect of inhibiting ATP synthesis on steady-state ATP levels. Since neurons largely rely on OXPHOS for ATP production ^33^, we blocked various components of the electron transport chain (ETC) *in vivo* with intraocular injection of pharmacological inhibitors (Fig2A). We first tested rotenone (ROT), an inhibitor of Complex I (CI) of the ETC. To our surprise, ROT injection resulted in precipitous ATP decline in a subset of RGCs while others were less affected (Fig2B,F). Injections in experimental replicates demonstrated that responses to ROT were reproducible, with some differences in severity of ATP depletion across samples (Fig2J). *Post hoc* immunofluorescence of these retinas revealed that αRGCs, especially αON-S, exhibited the greatest extent of ATP depletion (Fig2N,R). Similarly, inhibition of ETC Complex II (CII) and Complex III (CIII) by thenoyltrifluoroacetone (TTFA) and antimycin A (AA), respectively, also caused the greatest ATP decline in αRGCs and αON-S, albeit with different temporal dynamics, possibly due to differences in the pharmacodynamics of the inhibitors (Fig2C–T). Finally, inhibition of ETC Complex IV (CIV), the common endpoint of electron transport, by potassium cyanide (KCN) elicited ATP decline across the RGC population (Fig2E–U). Thus, specific metabolic inhibition blocking catabolic components CI-III that have anapleurotic pathways disproportionately depleted ATP in αRGCs and particularly αON-S. In contrast, inhibition of CIV, a severe metabolic chokepoint, caused energetic depletion across all RGCs. These results show that αRGCs not only have a lower homeostatic ATP set-point, but demonstrate greater sensitivity to metabolic challenge, and suggest that αRGCs may have reduced ATP production capacity or higher energetic demands.

### αRGCs have higher protein expression of mitochondrial ETC components

Lower baseline ATP and greater susceptibility to mitochondrial inhibition in αRGCs could be due to decreased expression of mitochondrial ETC components. Thus, we examined protein expression for mitochondria and key components of OXPHOS by performing quantitative confocal microscopy of retina wholemounts immunostained for TOM20, NDUFB8 (Complex I), SDHA (Complex II), UQCRC2 (Complex III), and ATP5A (Complex V) (**FigS1**). We used high-resolution volumetric confocal scanning of immunostained samples to 3D segment RGC somas labelled by RBPMS using Cellpose. Mitochondrial marker intensity was measured and SPP1^+^ cell masks were identified, allowing for automated immunofluorescence measurements in thousands of Alpha and non-Alpha RGCs in total. Despite lower baseline ATP and greater sensitivity to mitochondrial inhibitors, αRGCs exhibited higher staining intensity for TOM20, NDUFB8, SDHA, UQCRC2, and ATP5A, suggesting that αRGCs may be enriched for mitochondria and ETC components (**FigS1B**). Paradoxically higher mitochondrial expression in αRGCs indicates that their lower ATP is less likely due to decreased production capacity, potentially reflecting adaptation to a greater steady state ATP demand.

### Homeostatic ATP levels are stable under physiologic alterations in circuit activity

Lower ATP levels in αRGCs could be the result of greater ATP consumption, especially given that αRGCs are fast-spiking neurons ^6,7,34^. We first assessed our ability to alter circuit activity levels *in vivo*. In a separate cohort of mice, we expressed the Ca2+ FRET biosensor Twitch2B in RGCs, in which YFP/CFP ∝ [Ca2+] as a readout of RGC activity ^25,35^. As a positive control for activity modulation, we examined the effect of increasing or decreasing retinal circuit activity by intraocular injection of strychnine/bicuculline (Str/Bic) or NBQX/AP5 to block inhibitory glycine/GABA or excitatory glutamate receptors respectively. As expected, disinhibition by Str/Bic raised RGC Ca2+ levels (**FigS2A–E**) ^24^. Conversely, blocking glutamatergic input with NBQX/AP5 lowered RGC Ca2+ levels (**FigS2F–J**). These pharmacologic experiments demonstrate that Str/Bic and NBQX/AP5 altered RGC activity sufficiently to elicit sustained changes in intracellular Ca2+. We next tested the effect of physiologic activity alterations. Since 2-photon laser scanning microscopy is known to drive circuit activity in retinal explants and likely contributes to RGC activity *in v*ivo (Euler et al., 2019; Wang et al., 2021), we used the onset of image acquisition as an activity-altering light stimulus. In RGCs expressing Twitch2B, onset of image acquisition after 2 minutes of dark adaptation resulted in transient Ca2+ elevations in a subset of RGCs, consistent with our previous reports (**FigS2K–L**; Wang et al., 2021). To quantify this light-driven circuit activation, we calculated the absolute maximum change in Ca2+ during the first 10 seconds of scanning and found greater Ca2+ dynamics compared to apparent steady-state conditions reached after 2 minutes of continuous imaging (**FigS2M**).

**Figure 3.**
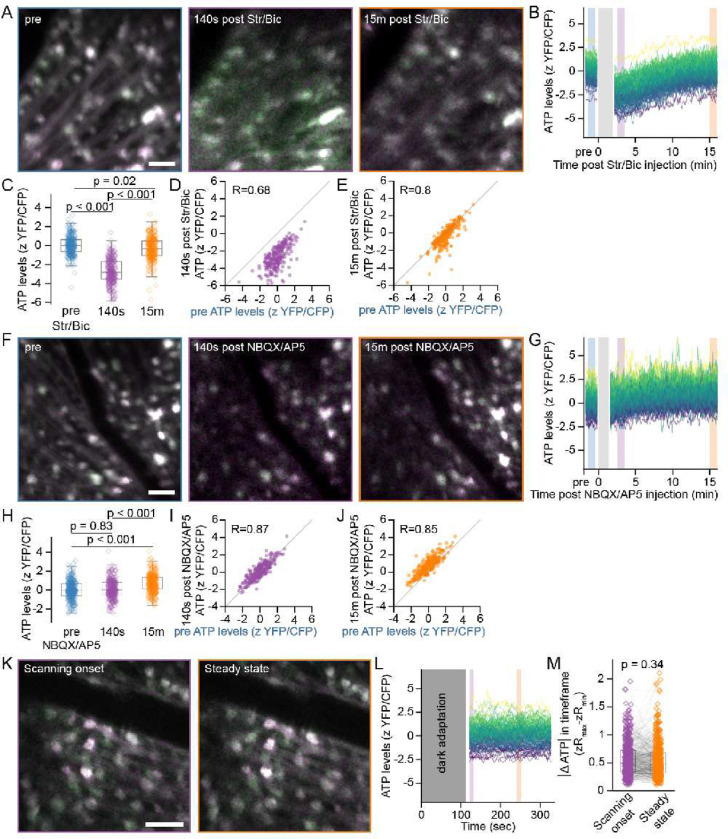
Effect of activity modulation on ATP in RGCs. (A) *In vivo* 2-photon aips of ATeam in the same retina imaged before and at the indicated time points after intravitreal injection of Str/Bic. (B) Traces of single RGC ATP levels tracked in an example retina in response to Str/Bic. Each line indicates an individual RGC. zScores are pseudocolored according to the values in the pre trace. (C) Scatter and box plots of RGC ATP levels before and at the indicated time points after intravitreal injection of the Str/Bic. Each point represents a single RGC. Colors indicate time points as represented by the boxed regions in (B). ANOVA with posthoc Tukey’s test throughout. (D-E) Scatterplots of ATP levels before and at the indicated time points after intravitreal injection of the Str/Bic (n=251 RGCs from 3 retinas). (F) *In vivo* 2-photon aips of the same retina imaged before and at the indicated time points after intravitreal injection of NBQX/AP5. (G) Traces of single RGC ATP levels tracked in an example retina in response to NBQX/AP5. (H) Scatter and box plots of RGC ATP levels before and at the indicated time points after intravitreal injection of the NBQX/AP5. (I-J) Scatterplots of ATP levels before and at the indicated time points after intravitreal injection of the NBQX/AP5 (n=282 RGCs from 4 retinas). (K) *In vivo* 2-photon aips of a retina dark adapted for 2 minutes and then imaged immediately after scanning onset (left) or 4 minutes later after reaching steady state (right). (L) Traces of single RGC ATP levels tracked in a dark-adapted example retina from the time point immediately after scanning onset. (M) Scatterpot of the change in ATP during the timeframe immediately after scanning onset or after reaching steady state indicated by the purple and orange boxes in (L) respectively (n=485 RGCs from 4 retinas). Kruskal-Wallis test with posthoc Dunn’s test. Scale bars = 50 μm.

We then performed these experiments in mice with ATeam-expressing RGCs. As expected, pharmacologically increasing activity by Str/Bic caused a population-wide decrease in ATP, which was followed by recovery toward baseline levels by 15 min (Fig3A–E), despite sustained calcium elevations at this time (**FigS2B–E**). Notably, ATP levels were reduced uniformly as the relative differences in ATP within the RGC population were maintained throughout, evidenced by the correlation of pre- and post-injection ATP levels (R_140s_ = 0.68, R_15m_ = 0.80) (Fig3D,E). Conversely, NBQX/AP5 resulted in a slight and delayed population-wide ATP elevation (Fig3F–J). Once again, relative ATP differences were preserved during this population-wide elevation (R_140s_ = 0.87, R_15m_ = 0.85) (Fig3I,J). Together, these results show that pharmacologic manipulation of retinal circuit activity alters ATP in all RGCs to a similar extent, possibly *via* affecting substrate availability in the local micro-milieu. We next examined the effect of physiologic activity changes on ATP levels. Despite demonstrating Ca2+ alterations during light-stimulation at the onset of 2-photon imaging, we did not observe changes in ATP levels in this timeframe (Fig3K–M). This finding suggests that the intracellular ATP pool in RGCs is robust enough to respond to acute physiological changes in activity-related demand that occur while processing light signals, possibly due to mechanisms in place for real-time metabolic homeostasis. This possibility is further supported by the observation of ATP recovery toward homeostatic levels after Str/Bic injection despite sustained elevations in Ca2+ (**FigS2B–E**, Fig3B–E).

### Selective ATP depletion after OXPHOS inhibition is driven by neuronal activity

**Figure 4.**
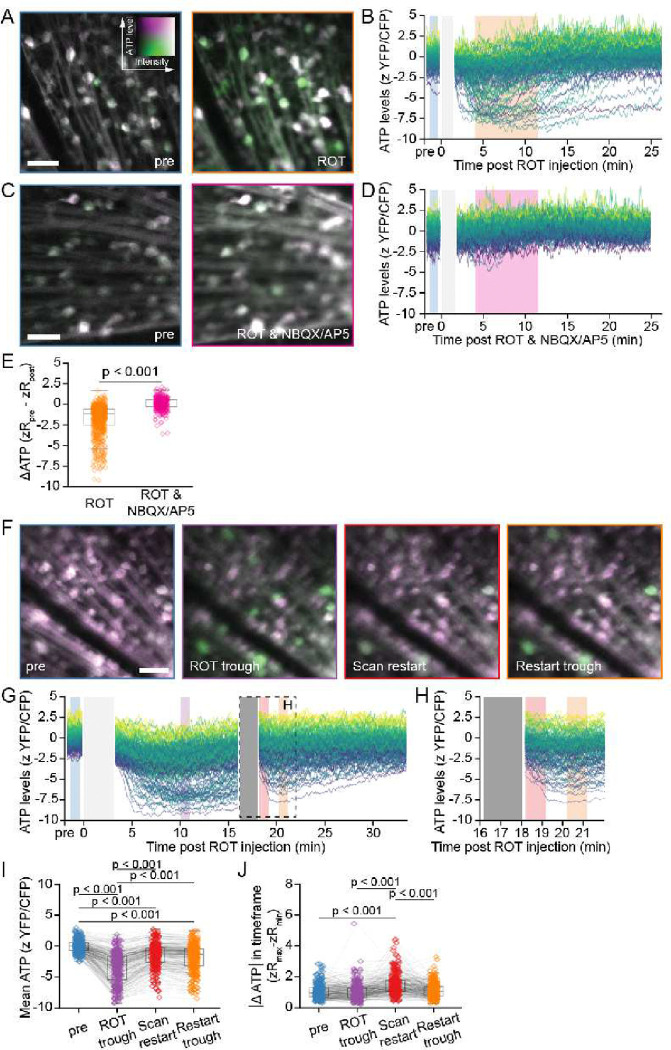
Role of activity on RGC ATP during mitochondrial inhibition. (A) *In vivo* 2-photon aips of ATeam in the same retina imaged before and after intravitreal injection of ROT. (B) Traces of single RGC ATP levels tracked in an example retina in response to ROT. Each line indicates an individual RGC. zScores are pseudocolored according to the values in the pre trace. (C) *In vivo* 2-photon aips of the same retina imaged before and after intravitreal injection of ROT & NBQX/AP5. (D) Traces of single RGC ATP levels tracked in an example retina in response to ROT & NBQX/AP5. (E) Scatter and boxplots of changes in ATP induced by ROT alone or ROT & NBQX/AP5. Orange and magenta regions in (B) and (D) respectively indicate time windows compared to pre (blue) to calculate ΔATP. Kruskal-Wallis test with posthoc Dunn’s test (ROT n=733 RGCs from 6 retinas (data replotted from Figure 2R), ROT & NBQX/AP5 n=349 RGCs from 4 retinas, rank sum test). (F) *In vivo* 2-photon aips of the same retina imaged before and after intravitreal injection of ROT with a period of dark adaption during the image acquisition. (G) Traces of single RGC ATP levels tracked in an example retina in response to ROT. Solid gray bar indicates dark adaptation period. (H) Zoom in of the example traces tracked in the boxed region of (G). (I) Scatter and box plots of individual RGC ATP in response to ROT injection and a period of dark adaptation. Colors match time windows indicated in (G), blue = pre, purple = post ROT trough, red = immediately after scanning restart, orange = trough after restart. Each point is an RGC and lines connect individual RGCs over time points. ANOVA with posthoc Tukey’s test (n=284 RGCs from 3 retinas). (J) Scatter and box plots of the change in ATP during the indicated time window calculated by subtracting the minimal observed level from the maximal observed level recorded within that time window. Scale bars = 50 μm.

Larger ATP decline in αRGCs, and especially αON-S, after CI-III inhibition could indicate higher energetic demand by these RGC types. Since αRGCs are also among the most strongly activated under limited light conditions ^36,37^, we tested whether neuronal activity may contribute to their selective ATP decline. We first measured ATP after ROT injection, or after ROT co-injected with NBQX/AP5 to simultaneously block neuronal activity (Fig4A–E). Compared to ROT-only control, co-injecting ROT with NBQX/AP5 significantly reduced the ATP decline in the RGC population (Fig4E).

Next, we confirmed the activity-dependence of ROT-induced ATP decline by interrupting our imaging, and thus pausing physiologic retinal circuit stimulation, in the presence of ROT. For RGCs that experienced ATP decline after ROT injection, 2-minute cessation of imaging caused ATP recovery toward baseline (Fig4F–H). Subsequent resumption of imaging led to a quick re-depletion. ATP measured during the minute immediately after imaging restart (“Scan restart”) was significantly recovered compared to the window prior to imaging hiatus (“ROT trough”) (Fig4I). Moreover, measuring acute dynamics during different timeframes, we found that ATP levels exhibited the greatest flux in the Scan restart window, reflecting rapid decline of cellular ATP upon laser scanning re-initiation (Fig4H,J). Importantly, after resumption of imaging, cells that again demonstrated ATP decline appeared to continue accumulating ATP at their original recovery rate (Fig4G). These dynamics indicate that blockade of ATP generation pathways may only manifest in neurons as ATP reductions when they are activated and thus consuming higher levels of ATP. Therefore, while physiologic neuronal activity was not sufficient to perturb steady-state ATP with OXPHOS intact, energy-limited conditions imposed by partial OXPHOS inhibition caused ATP stores to be sensitive to light stimulation. Together, these data show that neuronal activity is a main consumer of energy, but ATP is maintained at homeostatic levels by an intrinsic metabolic response requiring OXPHOS.

### Low baseline ATP is correlated with RGC resilience to axon injury

**Figure 5.**
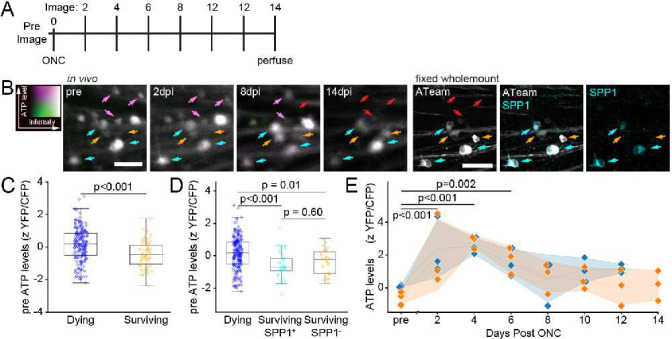
Relationship between RGC ATP and injury response. (A) Timeline of optic nerve crush survival experiments. (B) *In vivo* 2-photon aips of ATeam in the same retina imaged before and at the indicated time points after optic nerve crush. Cyan arrows indicate surviving SPP1^+^ RGCs, orange arrows indicate surviving SPP1^−^ RGCs, magenta to red arrows indicate dying RGCs. Scale bars = 50 μm. (C) Scatter and boxplots of homeostatic ATP levels in the pre-injury time point for RGCs that eventually died or survived the 14-day time lapse. Each point indicates an individual RGC (n=264 RGCs from 5 retinas, paired t-test). (D) Scatter and boxplots of dying RGCs with the surviving αRGCs identified by *post hoc* immunostaining of SPP1 separated out from the surviving cohort (n=264 RGCs from 5 retinas, One-way ANOVA with Tukey’s correction). (E) Line plot of ATP dynamics after optic nerve crush separated by dying (blue) and surviving (orange) RGCs. Lines represent mean, shaded area represents +/− sd, points represent retina sample means (n=188 RGCs from 3 retinas, ranked sum test of all RGCs at time point vs. pre).

Energetic stress can be a driver of neurodegeneration, and metabolic substrate supplementation can protect RGCs from degenerative models ^19,38–40^. Therefore, native differences in metabolic homeostasis could influence neuronal resiliency. Since we observed that natively resilient αRGCs had lower homeostatic ATP levels ^12^ (Fig1H), we asked whether a general relationship could exist between low homeostatic ATP and RGC resilience. We performed baseline ATP measurements of RGCs, followed by optic nerve crush (ONC) axon injury and longitudinally tracked RGC survival over 2 weeks (Fig5A). Surprisingly, RGCs that survived at 14 days post ONC had lower baseline homeostatic ATP levels compared to those that died (Fig5B,C).

Lower baseline ATP among the surviving population could indicate a general relationship between low ATP and resilience, or could reflect an enrichment of low-ATP αRGCs among the surviving cohort. To distinguish between these possibilities, we identified αRGCs in the surviving population using SPP1 immunofluorescence. As expected, surviving RGCs identified as αRGCs had lower mean baseline ATP compared to the population that did not survive (Fig5D). Interestingly, non-αRGCs surviving at 14 days similarly exhibited lower baseline ATP compared to the dying population (Fig5D). This finding suggests that low baseline ATP is correlated with RGC survival independent of RGC type.

### Population-wide ATP is transiently elevated days after ONC

Energetic depletion and subsequent AMPK activation have been implicated in traumatic and glaucomatous degeneration of RGCs ^38,41^. Thus, we examined day-by-day ATP dynamics in RGCs following ONC. Instead of expected reductions ^38,41,42^, we observed ATP elevations that began day 2 post ONC and peaked at day 4, before recovering toward baseline after 6 days post ONC (Fig5E). These longitudinal ATP dynamics indicate that metabolic changes occur in the days after ONC, possibly representing a protective response or sign of injury, and that cellular energetics become quiescent and stable later in the degeneration time course.

## DISCUSSION

By *in vivo* imaging ATeam, a well-established ATP biosensor ^5,23,43–45^ in combination with *post hoc* immunolabeling, we identified distinguishing energetic traits of physiologically distinct RGC types sharing a metabolic milieu. Previous studies have used biosensors to read out neuronal metabolic states *in vivo* but have not examined diversity across neurons ^5,44–47^. Our study demonstrates neuronal metabolic heterogeneity in distinct neuronal types, aided by high-throughput machine learning-based quantification workflows of neuronal metabolic traits observed dynamically at cellular resolution *in vivo*. By using RGCs as a model system for diversity within a closely related population of excitatory projection neurons, we have identified significant differences in energetic homeostasis of similar yet functionally divergent neurons within the same tissue environment. Here, our results demonstrate sustained ATP differences within the RGC population and attribute this heterogeneity, at least in part, to RGC types, providing *in vivo* confirmation of neuronal energetic differences theorized to exist decades ago ^48,49^.

While it is tempting to hypothesize that baseline homeostatic ATP differences are due to divergent neuronal activity, we do not suspect this to be the case. Strong circuit activation or inhibition by pharmacologic agents that disparately altered population-wide Ca2+ levels caused homogeneous population-wide ATP shifts that preserved baseline differences, possibly through altering substrate availability in the shared milieu thus affecting all RGCs to a similar extent. Rather, we expect that activity-independent pathways might account for baseline ATP variation. For example, the extents of mitochondrial uncoupling can influence ATP availability and may be salient in different neuronal types ^50,51^. Dynamic remodeling of the actin cytoskeleton and plasma membrane have also been proposed as significant sources of neuronal ATP consumption ^52–54^. It follows then that αRGCs, with characteristically large and ramified dendritic fields, may expend substantial amounts of energy to maintain their extensive cytoskeletal and membrane networks ^6,7^. Thus, the underlying basis of steady-state ATP variation in neurons remains a topic of investigation.

Regardless of the sources of baseline ATP differences, our study reveals the oversized role of activity in the neuronal energetic budget. Our experiments using mitochondrial inhibitors demonstrate that neuronal activity was a major source of ATP consumption, particularly in classically highly-active αRGCs, as reducing activity pharmacologically or by reducing light stimulus prevented ATP decline during OXPHOS inhibition. It is remarkable that OXPHOS appeared more dispensable in resting conditions, as pausing activity restored ATP toward baseline levels even in the presence of rotenone, and non-αRGCs that are less activated by 2-photon scanning did not experience as extensive ATP decline with most OXPHOS inhibitors. These findings provide *in vivo* validation of results in cultured neurons showing that inhibition of OXPHOS by oligomycin did not deplete ATP in resting neurons ^55,56^. Our data also address key controversies over the primary mode of ATP maintenance in active neurons, conclusively showing that glycolysis alone is not sufficient to maintain energetic homeostasis under signaling workloads *in vivo*, consistent with observations in cultured neurons and optic nerve explants ^55–57^.

Our results also shine light on the remarkable neuronal ability to maintain ATP homeostasis in the setting of rapidly shifting activity levels and consequent energetic demand. Consistent with studies using pharmacologic activity manipulation or high-frequency stimulation in cultured neurons, brain slice, optic nerve, and cerebral cortex ^43–45,55,57–59^, we observed population-wide ATP depletion due to Str/Bic. Even under these supraphysiological activation paradigms, ATP homeostasis was eventually restored. In our experiments, while Str/Bic induced a sustained Ca2+ increase out to 15 minutes after injection, ATP recovered steadily to near-baseline levels after an initial depletion, revealing a real-time bioenergetic response as has been observed *in vitro* ^55^. *In vivo* physiologic-range RGC activity changes induced by onset of 2-photon imaging altered cellular Ca2+ but caused no measurable ATP decline, consistent with studies using modest electrical stimulation of neurons *in vitro* ^44,56,60^ or whisker stimulation to activate somatosensory cortex *in vivo* ^44^. Multiple mechanisms have been implicated in this compensation, including glucose transporter translocation, AMPK activation, mitochondrial calcium uptake, activation of malate-aspartate shuttle, sodium pump activity, and ADP buildup ^44,55,60,61^. Our experiments provide *in vivo* proof for this real-time energetic adaptation at a cellular level, and suggest that OXPHOS is a requirement for this metabolic response.

We paradoxically found that RGCs that survived ONC had lower baseline ATP levels when examining the surviving cohort as a whole or when isolating the less intrinsically resilient non-αRGCs. Numerous studies in multiple neuronal contexts, including in models of RGC degeneration, have shown that neuronal death is associated with energy decline and can be ameliorated by inhibiting aberrant ATP consumption or supplementing energetic substrates ^19,38–40,62,63^. However, the lower levels of homeostatic ATP we measured in more-resilient RGCs are unlikely to be near metabolic failure. Estimates from whole rat retina indicate intracellular ATP concentrations ranging from 6 to 10 mM ^64–66^. Given that ATeam FRET ratios reach their maximum near 10 mM and the distribution of baseline ATP measurements across the RGC population represented roughly 20% of the ATeam dynamic range, we estimate differences between high and low baseline ATP levels of roughly 2 – 3.5 mM ^43,67^. While it is not possible to broadly conclude that low homeostatic ATP translates to increased resilience, we have shown that in the overall RGC population, cells with lower homeostatic ATP survived better. It is possible that lower maintained ATP levels during homeostatic conditions may confer a survival advantage when neurons later face degenerative energetic stressors, such as those potentially involved in the ATP changes we observed following RGC injury. Such a protective effect could occur by limiting the cumulative effect of harmful respiration-related factors like ROS ^68^, or through hormetic preconditioning to energy-limited states ^69^, permitting upregulation of mitochondrial biogenesis and other compensatory mechanisms.

## MATERIALS AND METHODS

Further information and requests for resources and reagents should be directed to and will be fulfilled by the lead contact, Philip R. Williams (prwillia@wustl.edu).

### Animals

All experiments involving animals follow protocols approved by IACUC at Washington University School of Medicine in St. Louis. Vglut2-Cre mice (Stock No. 028863) and C57BL/6J (Stock No. 000664) were purchased from Jackson Laboratories. Mice are housed in 12-hour light/dark cycle provided with food and water ad libitum. Male and female mice between 2–6 months of age were used for *in vivo* imaging, and between 2–3 months of age for ONC and quantitative immunofluorescence of mitochondrial protein expression.

### Intravitreal AAV injection

Intravitreal AAV injection was performed as previously described ^25^. Briefly, injection needles were fashioned from capillary glass micropipettes (Sutter #B150–86-10) with a pipette puller (Sutter Flaming/Brown Model P-97) and beveled to a sharp point using a custom beveling device ^70^. Needles were attached via mineral oil- (Fisher BP2629–1) filled tubing (McMaster Carr #1883T1, #1883T4) to a 50 μL Hamilton syringe (#80950). Mice were anesthetized by intraperitoneal injection of ketamine/xylazine cocktail (100 mg/kg ketamine, 10 mg/kg xylazine) and provided pre-op analgesia by subcutaneous injection of meloxicam (10 mg/kg). Sclera was punctured ~250 um posterior to the corneal limbus and ~1–2 μL of vitreous was withdrawn. 2 μL of AAV (Reagent List) was subsequently injected intravitreally. Antibiotic eye ointment was applied to the injected eye, and the animal was placed on a 37 degrees Celsius warming pad (McKesson #190147) and monitored for recovery from anesthesia. Mice were injected at least 2 weeks prior to experimentation for AAV transduction and expression.

### *In vivo* imaging

Transpupillary *in vivo* 2-photon imaging was performed as previously described ^24,25^. Imaging was performed using a multiphoton microscope (Hyperscope, Scientifica) through a long working distance 20X 0.4 NA Plan Apo NIR air objective (Mitutoyo #378–824-5) with excitation provided by an ultrafast Ti:Sapphire laser (Mai Tai DeepSee, Spectra-Physics) tuned to 850 nm. Laser power was controlled by a Pockels cell (Conoptics #350–80-02) and capped at 45 mW at the objective. Emission signal was split into YFP and CFP channels by a 505 long pass dichroic mirror and 535/30 and 480/40 band pass filters respectively and measured using GaAsP photomultiplier tube detectors (ChromoFlex, Scientifica). Images were acquired using ScanImage software (Vidrio Technologies).

Induction of anesthesia was achieved by ketamine/xylazine (as described above) and maintained using 0.5–1% inhaled isoflurane on 0.5 L/min room air. Pre-op meloxicam (see above) analgesia was provided for imaging experiments involving intravitreal injection.

Anesthetized mice were secured to the microscope stage using a head holder (Narishige SGM-4). Body temperature was maintained using a 37 degrees Celsius rodent heating pad (Stoelting 53800M). Pupils were dilated by atropine/phenylephrine eye drops (1% w/v atropine Sigma A0132, 2.5% w/v phenylephrine Sigma P6126). Clear lubricant eye gel (GenTeal, Alcon) was placed over the eye, and a #1.5 coverslip was held perpendicular to the microscope objective and brought into close proximity to the cornea in contact with the gel and secured in place by a slide holder (ThorLabs DH1). The mouse eye was subsequently aligned with the microscope objective, and the desired imaging area and focal plane were located under epifluorescence illumination at 488 nm. Imaging area was further refined under 2-photon illumination. Images captured were timeseries of 512 × 512 pixel raster scan frames of a single focal plane, acquired at a rate of 1.07 frames/second. For injection imaging experiments, a baseline timeseries was acquired, followed by intravitreal drug injection under a dissecting microscope illuminated with white light. Injected drugs were dissolved in a vehicle of 1:1 v/v mixture of dPBS and DMSO. The imaging area was then realigned under epifluorescence and 2-photon illumination, and the post-injection timeseries was acquired. Following imaging, mice were monitored for recovery as described above.

### *In vivo* image processing and quantification

Acquired images were saved as TIFF files of pixel intensity values generated by ScanImage with signal from the YFP and CFP detectors interleaved sequentially. Images were read using ScanImageTiffReader (https://vidriotech.gitlab.io/scanimage-tiff-reader/) and deinterleaved into separate 3-dimensional pixel intensity arrays for YFP and CFP channels. Background levels of each channel were measured as the median of pixel intensity values across time in corner regions of the image where the scan frame is clipped by the objective lens, and subtracted from the value of each pixel in that channel. Motion correction was accomplished by registering frames within a timeseries using Suite2p ^71^, and multiple acquisitions were aligned to the baseline image by scale-invariant feature transform (SIFT) ^72^ registration of their time- and channel- average projections and applying the transformation to each frame of the timeseries. Regions of interest (ROIs) encompassing RGC somas were generated on time- and channel- average projections using Cellpose ^28^ with manual correction of incorrect segmentations. Segmented ROIs of the same cells across multiple acquisitions were matched using the LAP tracker in TrackMate ^73,74^. Mean pixel intensity values within each ROI were measured across time for each channel, and high frequency temporal noise was filtered using a 20-frame moving average filter. ROIs containing < 100 pixels and with average YFP+CFP intensity < 50 across time were discarded. YFP/CFP values were then calculated for each ROI to obtain the FRET ratio and filtered YFP/CFP timeseries values for both baseline and post-injection images were z-score normalized to the baseline population mean and standard deviation. Code is available at https://github.com/zelunw/RGC-ATP

### Immunofluorescence and confocal microscopy

Mice were anesthetized with intraperitoneal injection of 250 mg/kg tribromoethanol (Sigma, T48402) and sacrificed by cervical dislocation. Enucleated eyes were fixed in 4% paraformaldehyde for 30 minutes at room temperature, and retina wholemounts were dissected and placed in 30% sucrose in PBS at 4 degrees Celsius overnight. Retinas were subject to 3 cycles of freeze-thaw on dry ice for antigen retrieval, washed and blocked in PBS + 0.2% TritonX-100 with 10% normal horse serum (Sigma, 158127) at room temperature for 1 hour, followed by incubation with primary antibody (see Key Resource Table) for 5 days and secondary antibody for 2 days at 4 degrees Celsius in the blocking buffer with 3 × 10-minute PBS washes between each step. Retinas were mounted on slides with #1.5 thickness coverglass in Vectashield (Vector Labs, H-1000–10) or Fluoroshield (Sigma #F6182) mounting medium with the ganglion cell layer facing up. Wholemount retinas were imaged using a Zeiss 20X 0.8 NA Plan Apo M27 air objective on a Zeiss LSM800 or LSM980 confocal microscope. Excitation and emission wavelengths for channels imaged were as follows: Blue: Ex 405nm, Em 410–450nm; Green: Ex 488nm, Em 500–553nm; Red: Ex 561nm, Em 570–632nm; Far-Red: Ex 639nm, Em 656–700nm; Near-Infrared: Ex. 730nm, Em 755–802nm.

### Immunofluorescence image analysis

For identification of RGC type, ATeam was imaged in the green channel and was used as reference for landmark-based registration of confocal and *in vivo* images using BigWarp ^31^. RGC type antibody positivity for each cell matched to an *in vivo* ROI was manually scored in maximum intensity z-projections of acquired confocal volumes.

For automated quantification of mitochondrial protein expression, one volumetric field of view was acquired with 0.2 μm z-step at a distance of ~1000 μm from the optic nerve head in each retina quadrant (dorsal, ventral, temporal, nasal). Using Cellpose, volumetric RGC soma masks were generated using the RBPMS-stained channel. Average fluorescence intensity of the mitochondrial proteins channel was measured in the middle 60% of z-slices of each cell ROI and z-score normalized to all ROIs measured in each volumetric field of view, and compared between RGC types. Samples using primary antibodies generated in mouse resulted in intense off-target labeling of endogenous immunoglobulins within retinal vasculature that were occasionally encompassed within ROIs; these ROIs were removed by discarding outliers with average fluorescence greater than or equal to the top quartile within the same image plus 1.5 times the interquartile range. ROIs were scored as SPP1-positive based on SPP1 channel fluorescence intensity greater than or equal to the top quartile within the same image + 1.5 times the interquartile range, measured in maximal z-projection of the image stack.

### Optic nerve crush

Optic nerve crush (ONC) surgery was performed as previously described ^24,26^. Briefly, mice were anesthetized by intraperitoneal injection of ketamine/xylazine and provided pre-op analgesia with subcutaneous meloxicam as described above. An incision was made in the lateral scleral conjunctiva, and the plane between the conjunctiva and sclera was developed by blunt dissection with Dumont #5/45 Forceps (Fine Science Tools #11251–35) and advanced toward the posterior pole of the globe. Upon visualization of the optic nerve, the tips of the forceps were positioned around the optic nerve 2 mm posterior to the globe and closed around the optic nerve for 5 seconds. Antibiotic eye ointment was applied over the ocular surface at the completion of surgery, and mice were placed on a heating pad and observed during recovery from anesthesia, with postoperative observation after 12 hours. Mice were killed 14 days after ONC as described above and eyes fixed with 4% paraformaldehyde for immunostaining.

### Longitudinal in vivo imaging and quantification

Prior to ONC, *in vivo* imaging was performed as described above for uninjured baseline. Following ONC, *in vivo* imaging was repeated every 2 days in the same region that was imaged prior to injury. For longitudinal cell tracking, time- and channel- averaged projections on each day were aligned by SIFT registration, and RGCs were tracked manually in TrackMate. Because caspase activity during apoptosis is known to cause cleavage of the linker region of the ATeam molecule, resulting in high fluorescence of dispersed CFP that does not correspond with cellular ATP concentration, cells with YFP/CFP ratios below 5 standard deviations of the pre-injury population mean during the course of degeneration were excluded from quantification and instead used to denote dying cells ^23^.

### Statistical analysis

Summary ATP of individual cells over specified timeframes and conditions were quantified as the area under the curve of the ATP z-scores across time (integrated using the composite trapezoidal rule) normalized to the duration of quantification. Group results are reported as mean +/− standard deviation or standard error of the mean where indicated. Boxplot overlays denote quartiles of the data points shown in scatter plots. Statistical comparisons were made using T-test, Wilcoxon rank-sum test, one-way ANOVA with posthoc Tukey’s test, or Kruskal-Wallis test with posthoc Dunn’s test where indicated. Statistical analysis was performed using SciPy, statsmodels, scikit-posthocs, and Graphpad Prism 9.
